# EFLM Working Group Accreditation and ISO/CEN standards on dealing with ISO 15189 demands for retention of documents and examination objects

**DOI:** 10.1515/almed-2023-0053

**Published:** 2024-01-30

**Authors:** Pika Meško Brguljan, Marc H.M. Thelen, Francisco A. Bernabeu-Andreu, Christos Kroupis, Guilaine Boursier, Ines Vukasović, Edward Barrett, Duilio Brugnoni, Maria Lohmander, Luděk Šprongl, Tatjana Vodnik, Irina Ghiţă, Florent Vanstapel, Michel Vaubourdolle, Willem Huisman

**Affiliations:** University Clinic for Respiratory and Allergic Diseases, Golnik, Slovenia; Department of Laboratory Medicine, Radboud University Medical Centre, Nijmegen, the Netherlands; Foundation for Quality Assurance in Laboratory Medicine (SKML), Nijmegen, the Netherlands; Servicio de Análisis Clinicos - H.U. Puerta de Hierro Majadahonda, Madrid, Spain; Department of Clinical Biochemistry, Attikon University General Hospital, Medical School, National and Kapodistrian University of Athens, Haidari, Greece; Department of Genetics, Rare Diseases and Personalized Medicine, CHU Montpellier, Univ Montpellier Montpellier, France; Department of Clinical Chemistry, Sestre Milosrdnice University Hospital Center Zagreb, Croatia; Consultant Clinical Biochemist, Limerick, Ireland; Clinical Chemistry Laboratory - Spedali Civili, Brescia, Italy; Laboratoriemedicin NU-Sjukvården Trollhättan, Sweden; Clinical Laboratory, Hospital Kladno Kladno, Czech Republic; Center of Medical Biochemistry, Clinical Center of Serbia, Belgrade, Serbia; Synevo Central Lab Clinical Trials, Iflov County, Chiajna, Romania; Laboratory Medicine, University Hospital Leuven, Leuven, Belgium; Department of Public Health, Biomedical Sciences Group, Catholic University Leuven, Leuven, Belgium; Département de Biochimie, Hôpital Saint-Antoine – AH-HP, Paris, France; Consultant European Specialist in Clinical Chemistry and Laboratory Medicine, The Hague, the Netherlands

**Keywords:** EFLM, failure-mode-effects-analysis (FMEA), ISO 15189, quality management, retention time, risk assessment

## Abstract

Many aspects of the activity of a medical laboratory have to be documented so as to facilitate the maintenance of the ongoing quality of service. As a consequence, many documents, forms and reports are generated. The retention time for each of these has to be specified. In addition to medical laboratory reports as part of the patient’s medical record, the medical laboratory has to retain many documents and specimens according to national legislation or guidance from professional organizations, if these exist. If not, the laboratory management needs to define a retention schedule, which shall define the storage conditions and period of storage, according to ISO 15189:2022 requirements for retention of general quality management documents and records. The EFLM Working Group on Accreditation and ISO/CEN standards provides here a proposal on retention periods of documentation and specimens based on a failure-mode-effects-analysis (FMEA) risk-based approach, a concept of risk reduction that has become an integral part of modern standards.

## Introduction

Many aspects of the activity of a medical laboratory have to be documented so as to facilitate the maintenance of the ongoing quality of service [1], [2], [3], [4], [5]. As a consequence, many documents, forms and reports are generated. The retention time for each of these has to be specified in the laboratory’s Quality Management System [1], [2], [3].

Medical laboratory reports are part of the patient’s medical record. They shall be retained for the time period stated in the national legislation. They may also remain available in either the laboratory information system or archived as hard copy for a specified time period, which can be more than 100 years (lifelong) for certain specific examinations. If specific retention periods are not defined, the laboratory management needs to define a retention schedule for the different type of documents, forms, records and specimens. This schedule shall define the storage conditions (e.g. paper or electronic for documents) and the duration of storage according to ISO 15189:2022.

According to ISO 15189:2022 [2] the documentation can be in any form or type of medium, providing it is readily accessible and protected from unauthorized changes and undue deterioration. Facilities shall provide a suitable environment for storage of records to prevent damage, deterioration, loss or unauthorized access. For some records, especially those stored electronically, the safest storage may be on secure media and an offsite location (or even cloud applications with adequate and satisfactory security). The length of time that records are retained may vary, however, patient results reports shall be retrievable for as long as is medically relevant or as required by regulation. There is also a need of Accreditation bodies for guidelines on requirements for retention time issues.

A questionnaire prepared by the European Federation of Clinical Chemistry and Laboratory Medicine (EFLM) Working Group Accreditation and ISO/CEN standards (WG-A/ISO) was issued in 2011 in order to assess the current practices in the countries represented by member societies of the EFLM. The results illustrated a wide dispersion in practices and led to the preparation of this document. In what follows we will illustrate the appropriateness of a failure-mode-effects-analysis (FMEA) approach to select retention times for elements of the laboratory documentation system. For patient records and retention of examination objects more often regulators will have set retention times.

## Materials and methods

### Questionnaire

An extensive questionnaire about all the documents listed in clauses 4.13 and 5.7.2 of the ISO 15189:2012 standard [1] on the requirements for the retention of laboratory records and diagnostic material was sent to EFLM member societies in 2011.

### Risk analysis

Originally developed for the manufacturing industry, risk management principles [6, 7] have applications in identifying and controlling sources of laboratory error (CLSI EP18-A2, ISO 22367, CLSI EP23-Ed2) [8], [9], [10]. Based on risk management analysis and by using the FMEA technique, reasonable retention times can be chosen and prospective risk assessment can be used to establish if they are indeed sufficient.

In FMEAs, the probability of a risk (P) is rated first:

**Table:** 

Probability (P)	
Rating	Meaning
A	Extremely unlikely (virtually impossible or no known occurrences on similar products or processes, with many running hours)
B	Remote (relatively few failures)
C	Occasional (occasional failures)
D	Reasonably possible (repeated failures)
E	Frequent (failure is almost inevitable)

Then, the severity (S) is rated:

**Table:** 

Severity (S)	
Rating	Meaning
I	No relevant effect on reliability or safety
II	Very minor, no damage, no injuries, only results in a maintenance action (only noticed by discriminating and demanding customers)
III	Minor, low damage, light injuries (affects very little of the system, noticed by average customer)
IV	Moderate, moderate damage, injuries possible (most customers are annoyed, mostly financial damage)
V	Critical (causes a loss of primary function; loss of all safety margins, one failure away from a catastrophe, severe damage, severe injuries, max one possible death)
VI	Catastrophic (product becomes inoperative; the failure may result in completely unsafe operation and possible multiple deaths)

Then, probability and severity are combined to grade the risk:

**Table:** 

Risk grade (P*S)						
Severity						
Probability	I	II	III	IV	V	VI
A	Low	Low	Low	Low	Moderate	High
B	Low	Low	Low	Moderate	High	Unacceptable
C	Low	Low	Moderate	Moderate	High	Unacceptable
D	Low	Moderate	Moderate	High	Unacceptable	Unacceptable
E	Moderate	Moderate	High	Unacceptable	Unacceptable	Unacceptable

Finally, the detection rate is assessed. In the case of retention times, this is the chance that the need for consultation of documents or retesting of examination objects is recognized before they are discarded.

**Table:** 

Rating of detection	Meaning
1	Certain – fault will be caught immediately
2	Almost certain
3	High
4	Moderate
5	Low
6	Fault is undetected by operators or maintenance personnel

The lower the chance of timely detection (the higher the rating ranked from 1 to 6) the lower the risk category that can be accepted.

## Results and discussion

A wide dispersion in retention times for the documents listed in clauses 4.13 and 5.7.2 of the ISO 15189:2012 standard [1] on general quality documentation, personnel records, equipment records, analytical results and raw data was evident in the responses received from nine EFLM member national societies (29 % response rate) (W. Huisman, Presentation at EA (European Accreditation) meeting: “Results of EFLM WG ISO/CEN Accreditation survey on Requirements for the retention of laboratory records and diagnostic material”, 2011 – personal communication) (Supplementary Table 1) [11]. In the new version of the ISO 15189:2022 standard the issues on retention times are addressed in clauses 8.4.3 and 7.4.2 [2]. In comparison with ISO 15189:2012, the new version ISO 15189:2022 is less prescriptive on documentation need, including retention issues. The laboratory shall specify the retention times. In addition to requirements, the retention times can be chosen based on identified risks [2].

The low response rate may be indicative of the difficulty to make an inventory of practices on a multitude of different items. This is also reflected by the diversity of the responses. We encountered specific legal requirements for genetic data in Germany, based primarily on the concept of privacy, sovereignty e.g. right-to-not-know and the concept of genetic exceptionalism.

The need for harmonization of retention time is obvious. Hence, this EFLM WG A/ISO proposal was prepared.

### Needs of accrediting bodies

The retention time in many countries (W. Huisman, Presentation at EA (European Accreditation) meeting: “Results of EFLM WG ISO/CEN Accreditation survey on Requirements for the retention of laboratory records and diagnostic material”, 2011 – personal communication) [11], [4, 5, 12] is usually a fixed period from the time that the record was created (e.g. a specimen was received), but may be a fixed period following a defined event (e.g. after completion of the study, after termination of employment for a personnel record, etc.).

Since it is difficult to decide which retention times are reasonably useful [12], regulators and assessors tend to demand long retention times in order to not to discard document or material too early. Unlimited retention of documents, however, conflicts with lean management and conscientious use of resources.

One of the approaches used to rationalize the default retention time for quality documents or traceability proofs and to make auditing by regulators possible is to use the interval between two audits, as suggested by some accreditation bodies, e.g. COFRAC, the French Committee for Accreditation, recommends at least twenty-four months by default [13, 14]. According to ISO-15189:2022, fixing retention times is the prerogative of the laboratory. In addition to requirements, retention times, can be chosen based on identified risks. Legal liability concerns regarding certain types of procedures (e.g. histology examinations, genetic examinations, pediatric examinations) can require the retention of certain records for much longer times than for other records [2].

### Failure-mode-effects-analysis

An approach is to strive for rational principles that provide direction to useful retention times. Due to the rapid changes in laboratory medicine in the last few years and the implementation of the concept of risk reduction, we propose to use an approach based on the risk management concept in order to establish retention times that are long enough to prevent risk of ‘*regret of discard*’, and short enough to be practical and responsible with resources.

In order to establish useful retention times, we first have to consider the possible reasons for the use of documents and materials after the period of their original use. When different reasons for retention co-occur, the reason with the longest need for retention, determines the minimal retention time.

### Examples with possible reasons for retention of related documents

#### Review of the original request form

(a) Consultation initiated by the laboratory upon authorization or release of the result.
(b) Consultation initiated by the requesting physician upon use of the reported results.
(c) Complaints of the requesting physician upon use of the reported results.

From these possible reasons for retention, the reason with the longest possible effect is selected in order to determine the retention time. In this case, this is reason (b). Reason (a) has the shortest possible effect; once the report has left the laboratory, only reasons initiated by the requesting physician will lead to review of the request. Reason (b) and (c) generally coincide. From the side of the requesting physician, complaints are mostly initiated at the first moment of ‘consumption’ of the reported results of the request. Consultation, however, may be initiated at a later time, for instance, the time when the requesting physician sees the patient to discuss the results. Therefore, a rational retention time for the original request (form) can be the maximum time between the request and the next patient visit.

If we assume this period to be maximally eight weeks, we can perform an FMEA risk analysis to establish the risk and evaluate whether this period is too short.

**Table:** 

FMEA	
Error type	Discarding the original request form eight weeks after reporting the results, but before requesting physician has contacted laboratory for consultation about the results in the light of the original request
Probability, P	B (remote)
	In most cases patient is seen within eight weeks after request is presented to lab
Severity, S	II (very minor)
	Even without the original request form available consultation about the results can be performed
Risk grade (P*S)	Low
Detection	6 (undetected)
	The laboratory cannot prevent discarding the form when patient is not seen within eight weeks after presentation of request
Acceptability	Highly acceptable

Since consultation is relatively independent of the availability of the request form, we can also perform the FMEA for the chance that the request is discarded before the requesting physician has made a complaint about the laboratory service upon the request (reason c).

**Table:** 

Error type	Discarding the original request form eight weeks after reporting the results, but before the requesting physician has contacted laboratory for consultation about the results in the light of the original request
Probability, P	B (remote)
	In most cases report is seen within eight weeks and in most cases, there are no complaints
Severity, S	III (minor)
	Without the request form available discussions about its content can be tedious, but it could never represent any danger
Risk grade (P*S)	Low
Detection	6 (undetected)
	The laboratory cannot prevent discarding the report when it is not seen within eight weeks after initial issue of the report
Acceptability	Highly acceptable

#### Review of results by laboratory staff

(a) Plausibility check by laboratory before reporting results
(b) Plausibility check by laboratory after reporting results

Since the availability of a newer result can induce doubt about the accuracy of a previous result, it seems reasonable to retain all original data necessary to reconstruct a result at least for as long as in most cases a newer result of that test is being reported. This means that e.g. for test X after one year in most cases, results have been followed up by a newer result. Only data of tests with results that do not tend to change during life-time are not followed by a second test and are kept forever (DNA genetic tests).

Data needed to be retained in order to reconstruct the reported result:

– Reagent batch numbers
– Reagent inserts
– Calibration data
– Result raw data
– QC data
– Identification of operators involved
– Education status of all personnel involved

**Table:** 

FMEA	
Error type	Discarding the original data necessary to reconstruct the original result before a newer result comes in, but after one year
Probability, P	B (remote)
	In most cases plausibility can be established before newer result is available. In most cases a newer result is either available within one year, or not useful for evaluation of the plausibility of the previous result
Severity, S	III (minor)
	When probability is doubted upon availability of newer results but after one year it is doubtful whether results older than one year can be reasonably doubted
Risk grade (P*S)	Low
Detection	4 (moderate)
	The laboratory can have a separate retention period for tests where retention for longer period seems useful, e.g. DNA results
Acceptability	Highly acceptable

In this way, all different documents, forms and records can be analyzed. An efficient way is to make a number of timescales, e.g. eight weeks for raw data from the instruments, details of the persons who received the sample and handled it in the laboratory; the time between assessment control visits by the accreditation body (in most countries one year) can be used for internal quality control records, complaint handling and related aspects. The time between reassesments of accreditation (four to five years in most countries) could be used for external quality control records.

### Retention of samples and specimens

The laboratory shall define the length of time for clinical samples to be retained. Retention times depend on the nature of the sample, its stability, the typical frequency of performing the test or examination and any other relevant requirements, such as the ease of collecting the sample (e.g. pediatric examinations) or (e.g. genetic testing, oncology and histopathology), often also determined by regulations and laws.

### Example of retention times

Sample retention times for documentary elements of the quality system and for samples and examination objects are summarized in Supplemental Material, Appendices A and B, respectively. Retention times of sample risk analysis shall not be read as guidance. Comparing with the survey results, in most cases, risk assessment has shown as more lean approach in retention times requirements [11].

## Conclusions

Medical laboratories document their activities among other things to ensure the ongoing quality of service. The EFLM WG-A/ISO suggests that medical laboratories follow the risk-based approach set out in this document to define appropriate and specific retention schedules for documents, forms, records and specimens.

Sample retention times for documentary elements of the quality system and for samples and examination objects, which are summarized in Supplemental Material, Appendices A and B, respectively, shall not be read as guidance. It is of the utmost importance to have your own individual lab-oratory and clinical-use-specific risk assessment. Any legal regulations in force take precedence over our proposal on how to choose appropriate retention times. It was not our ambition to summarize legal regulations in force (e.g. in the fields of genetic testing or blood banking).

## Supplementary Material

Supplementary Material Details

Supplementary Material Details

Supplementary Material Details
