# Supplementary material for: EFLM Working Group Accreditation and ISO/CEN standards on dealing with ISO 15189 demands for retention of documents and examination objects
Source: Adv Lab Med. 2024 Jan 30;5(2):103–8. doi: 10.1515/almed-2023-0053 (PMC11206180; doi:10.1515/almed-2023-0053)
Supplement: Supplementary file 1 — Supplementary Material Details [file j_almed-2023-0053_suppl_001.docx]

Appendix A:

Sample retention times for elements of the documentary quality system

According to ISO 15189 the following general quality management documents and records should be retained. The medical laboratory shall define its own retention times for these documents, except when legal requirements are imposed.

General quality management documents:

The retention periods are presented as an example (in years, if not otherwise specified):

- Legal entity - at least five years

- Quality manual – five years

- Procedures - five years

- Documents for effective planning, operation and control of laboratory’s processes - five years

- Copies of applicable regulations, standards and other normative documents – five years

Records:

The retention periods are presented as an example (in years, if not otherwise specified):

- Supplier selection and performance, and changes to the approved supplier list – five years

- Staff qualifications, training and competency records (personnel records)-employment plus one year

- Examination requests - eight weeks

- Records of receipt of samples in the laboratory - eight weeks

- Information on reagents and materials used for examinations (e.g. lot documentation, certificates of supplies, package inserts, etc.) – five years

- Laboratory work books or work sheets – one year

- Instrument printouts and retained data and information – one year

- Examination results and reports – five years

- Instrument maintenance records, including internal and external calibration records - for the life of the equipment plus one year

- Calibration functions and conversion factors- five years

- Internal Quality Control records -one year

- Incident records and actions taken – five years

- Accident records and actions taken – five years

- Risk management records – five years

- Nonconformities identified and immediate or corrective actions taken – five years

- Preventive actions taken – five years

- Complaints and actions taken – five years

- Records of internal and external audits – five years

- Inter-laboratory comparisons of examination results – five years

- Records of quality improvement activities – five years

- Minutes of meetings that record decisions made about the laboratory’s quality management activities – five years

- Records of management reviews – five years

- Reports-(as part of patient documentation, depends on national regulations and legislation)
