# Supplementary material for: EFLM Working Group Accreditation and ISO/CEN standards on dealing with ISO 15189 demands for retention of documents and examination objects
Source: Adv Lab Med. 2024 Jan 30;5(2):103–8. doi: 10.1515/almed-2023-0053 (PMC11206180; doi:10.1515/almed-2023-0053)
Supplement: Supplementary file 2 — Supplementary Material Details [file j_almed-2023-0053_suppl_002.docx]

Appendix B:

Sample retention times for clinical samples or specimens

The medical laboratory shall define its own retention times for clinical samples and specimens, except when legal requirements are imposed. Retention times depend on the nature of the sample, its stability, the typical frequency of the measurement or examination and any other applicable and relevant requirements or special regulations (e.g. genetic, pediatric examinations).

- General (including clinical chemistry, immunology): seven days from date of receipt (or until two days after the date of the issued report, if this is longer). The storage conditions shall be appropriate and retrieval shall be reliable (it is often acceptable to discard urine specimens submitted for dip-stick testing, plasma for routine coagulation tests, citrated blood for BSE and liquor for cell counting much earlier).

- Hematology: Blood samples: seven days (for purpose of identification and traceability); plasma samples for haemostasis testing: seven days (for purpose of identification and traceability); special tests: frozen one month; blood films: one month; bone marrow slides: 3 years.
